# Fraudulent ID using face morphs: Experiments on human and automatic recognition

**DOI:** 10.1371/journal.pone.0173319

**Published:** 2017-03-22

**Authors:** David J. Robertson, Robin S. S. Kramer, A. Mike Burton

**Affiliations:** Department of Psychology, University of York, York, United Kingdom; University of Nottingham, UNITED KINGDOM

## Abstract

Matching unfamiliar faces is known to be difficult, and this can give an opportunity to those engaged in identity fraud. Here we examine a relatively new form of fraud, the use of photo-ID containing a graphical morph between two faces. Such a document may look sufficiently like two people to serve as ID for both. We present two experiments with human viewers, and a third with a smartphone face recognition system. In Experiment 1, viewers were asked to match pairs of faces, without being warned that one of the pair could be a morph. They very commonly accepted a morphed face as a match. However, in Experiment 2, following very short training on morph detection, their acceptance rate fell considerably. Nevertheless, there remained large individual differences in people’s ability to detect a morph. In Experiment 3 we show that a smartphone makes errors at a similar rate to ‘trained’ human viewers—i.e. accepting a small number of morphs as genuine ID. We discuss these results in reference to the use of face photos for security.

## Introduction

The use of fraudulent ID is a significant societal problem. In this study we examine one potential route to fraud: the use of a manipulated facial image in a photo-ID. The extensive psychological literature on face processing suggests a number of vulnerabilities that could be exploited by people wishing to deceive ID checkers (human or machine), and here we consider the use of graphical morphing.

Passports carry a photo of the bearer, and are the target of fraud across many countries. To deter this, anti-counterfeit measures have become increasingly sophisticated, for example through the inclusion of invisibly printed patterns detectable only under particular artificial illumination. As these measures are difficult to circumvent, criminal attacks on passport systems are increasingly focussing on ‘Fraudulently Obtained Genuine’ documents, or FOGs [1–2]. These are real documents issued to fraudulent applicants. This means that passport-issuing authorities must address the challenge of detecting incorrect details on applications—including ID photographs.

Passport officials checking IDs may have access to other photos of the applicant, for example images from earlier applications or other official documents such as driving licences. However, checking two different images of an unfamiliar person is known to be very difficult and prone to error [3–4]. Furthermore, comparing a real person to a photo is just as difficult as comparing two photos [5–7], and the problem becomes harder as the time between the photos increases, as in passport renewals [8]. This difficulty is not confined to novice viewers: White et al [9] demonstrated that a group of working passport officers were no better at face matching than a group of untrained students. So, it seems clear that a fraudulent application with a false photograph may not be easy to detect.

In the experiments below, we address a recent phenomenon which could make fraudulent photos even harder to detect—the use of face morphs [10–11]. In one form of attack, a complicit passport owner submits a renewal application with the photo of a second person. The aim is to find pairs of people (legitimate owner and second person) who look sufficiently alike that a discrepancy will not be noticed—i.e. to exploit the difficulty of this task for a viewer. In a development of this attack, a photo may be submitted which is a morph between the legitimate owner and the second person—with the aim that the resulting image looks sufficiently like both parties to become acceptable photo-ID for either [11]. Graphical morphing is now widely available, and allows for generation of a near-continuous sequence of images forming intermediate steps between one image and another. The central point of this continuum could be said to contain equal amounts of each original photo.

Psychological evidence, at first glance, suggests that this may not be a very efficient form of attack. Early research with image morphing suggested that perception of identity is categorical, i.e. perception of identity does not blend along the continuum. Instead, there is a boundary somewhere between the two original images, and identity is seen clearly for one of the individuals either side of that boundary [12]. However, this early research was conducted with *familiar* faces. The situation is more nuanced with unfamiliar faces [13–15], and categorical perception seems to rely on explicit training of the two identities before testing [16]–training which would not be available to viewers processing a passport application. Furthermore, in many categorical perception tasks, participants are asked whether a particular image represents person A or person B. This is not the problem facing a passport checker, who instead has to decide whether a single person is or is not legitimate.

In Experiments 1 and 2 we assess the extent to which computer generated morphs are accepted as a genuine face photo, when participants are unaware (Experiment 1) and aware (Experiment 2) of the use of face morphs in passport fraud. Advances in technology mean that automatic face recognition systems are now common, both in high-security settings like border-gates, and also in personal electronics, e.g., for access to mobile phones. In Experiment 3 we test a highly popular mobile phone, equipped with face recognition security, and ask whether it can withstand attack using a morphed image.

## Experiment 1

In this experiment, we examined the extent to which computer generated face morphs are acceptable as genuine matching ID. Across a set of trials, viewers were asked to decide whether a photo-ID (passport) matched a second face image or not. In fact, sometimes the faces matched, sometimes they did not, and sometimes the ID photo was a morph between a matching and non-matching face. As the fraudulent use of face morphs is a recent development, we wanted first to assess identity-checkers’ performance when they were not told to expect that some of the images would have been graphically manipulated, and so no mention was made of this possibility in the instructions. We anticipated that viewers would quite frequently be willing to accept morphed images as true matches, particularly if they contained a substantial proportion of the paired identity. However, we were particularly interested to observe acceptance rates at the 50/50 level (presumably the most useful in real fraud). We were also interested to observe whether acceptance rates declined smoothly (as the match image contained less and less of the target) or exhibited a step function signifying categorical perception, as described above.

### Method

#### Ethics statement

This study was approved by the Ethics Committee of the Department of Psychology, University of York. All participants gave written informed consent.

#### Participants

Twenty eight participants (25 female) with a mean age of 20 years (SD = 3, Range = 18–31) were recruited from the University of York Department of Psychology. All participants were naïve to the purpose of the study and received a course credit or monetary payment for their participation.

#### Stimuli and apparatus

**Fig 1:**
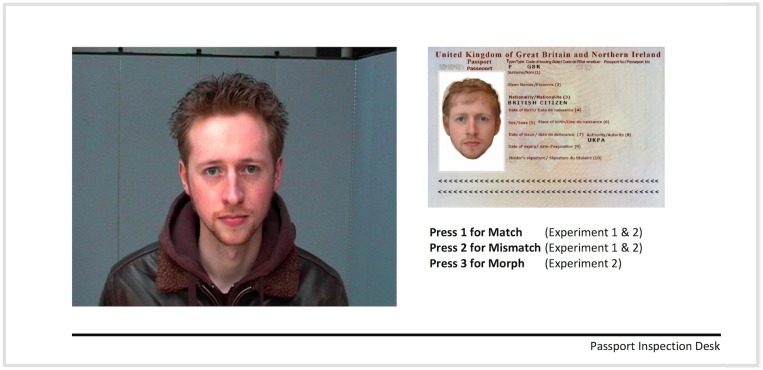
Photographic comparison. An example trial in which the passport photo contains 50% of the target identity (left) and 50% of the fraudster’s image (right, embedded in the passport frame). The individuals shown in Fig 1 have given written informed consent (as outlined in PLOS consent form) to publish these images.

Facial images were taken from a standardised test of face matching, the Glasgow Face Matching Test, GFMT [17]. That test comprises pairs of face photos taken with two different cameras on the same day—sometimes showing the same person, and sometimes different people. In the original test, image pairs are cropped around the face, and presented side-by-side. To create stimuli for this experiment, we presented the full, uncropped image for one of the pair, at a size of 14cm x 10.5cm. The second photo was embedded in a UK passport frame, using the dimensions of real UK passports (frame: 8.8cm x 12.5cm; photo 3.5cm x 4.5cm). Fig 1 shows an example.

**Fig 2:**
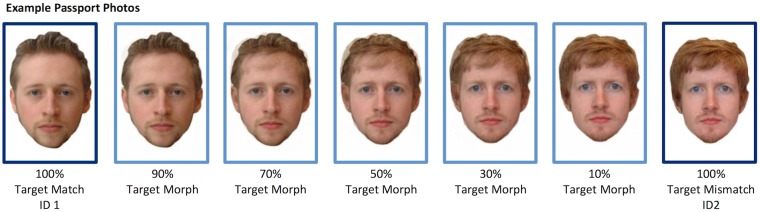
Morph levels used in passport frames for Experiments 1 and 2. The individuals shown in Fig 2 have given written informed consent (as outlined in PLOS consent form) to publish these images.

Stimuli for the experiment comprised images of forty nine identities (19 female) from the GFMT. For each target face, match and mismatch images were drawn from the test. We also created images in which the photo to be embedded within a passport frame (Fig 1) was a morph between the match and mismatch face. Image manipulation software Psychomorph [18] was used to create five morphed images per pair of identities; the images were transformed from one ID to the other in steps of 20%. Consequently, for each identity there was an uncropped GFMT target image and 7 passport photos; 100% match, 100% mismatch, and morphs with ratios of match/mismatch: 90/10; 70/30; 50/50; 30/70; 10/90. Fig 2 shows an example. All images were presented on a 12-inch Hewlett Packard laptop using E-Prime 2.0.

#### Procedure

Participants were presented with two images on each trial, as shown in Fig 1. The uncropped target image always appeared on the left side of the display and the passport photo always appears on the right. Each participant was told that they were taking on the role of a passport inspection officer at an airport and that on each trial they would encounter a ‘traveller’ (target image) bearing a passport (passport frame and photo). They were instructed to decide whether or not the traveller’s face matched their passport photo (Press ‘1’) or did not (Press ‘2’).

Each participant completed 49 trials (7 of each morph level, presented in a random order). Each trial remained onscreen until response. The experiment was counterbalanced such that across participants, each target image was paired with each of its morph-level passport photos on an equal number of occasions.

### Results and discussion

**Fig 3:**
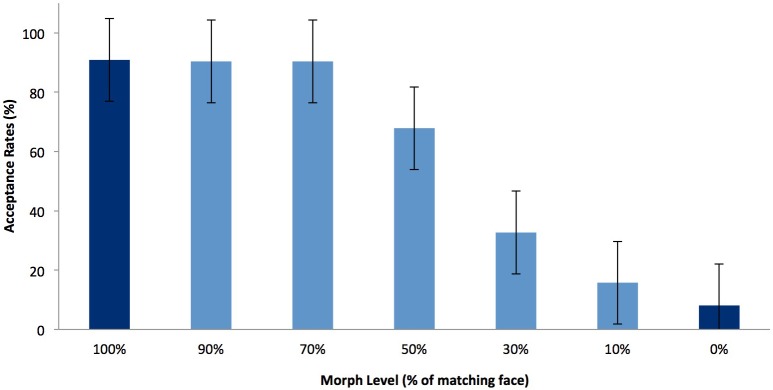
Mean passport image acceptance rates across morph levels for Experiment 1. (Error bars denote standard error.)

Fig 3 shows the acceptance rates for passport photos across the different levels of morphing. Error rates for the endpoints (100% match and 100% mismatch) are low (9% and 8% respectively) and consistent with average population performance on the GFMT (Long form, [17]). Single-factor ANOVA across conditions shows a strong influence of morph level (F(6,162) = 181.54, p < 0.001, *η*^2^ = .87). Tukey HSD comparisons between morph levels show no significant difference in acceptance rates between 100%, 90% and 70% morphs, but a significant drop between levels 70% and 50% (F = 33.75, p < 0.01), with further significant drops in steps 50% to 30% (F = 82.51, p < 0.01), and 30% to 10% (F = 19.03, p < 0.01).

While 50/50 target morphs were accepted as genuine matches less frequently than genuine photos, they were nevertheless accepted at a surprisingly high level. An acceptance rate of 68% suggests that this type of image, when used as fraudulent ID, might sometimes serve the purpose of providing acceptable documentation for two different people. However, note that in this experiment, we did not alert the participants to the possibility that some of the photos may have been graphically manipulated. We assumed that viewers would reject as ‘fraudulent’ any image that they noticed had been manipulated—however, it is possible that they made more sophisticated judgements than this, accepting manipulated images as long as they retained some likeness of the second image. In the following experiment, we repeat the matching task, but instruct viewers to look out for these manipulated images.

## Experiment 2

This experiment used the identical stimuli and procedure as Experiment 1, with the exception that this time, participants were briefed in advance about the possibility of ‘fraudulent’ ID using graphics morphing, and during the test were given an extra response option. When trying to match the face photos (see Fig 1), they now had the options to respond ‘match’, ‘mismatch’ or ‘morph’. We examined whether this prior warning would affect their accuracy in judging the photo ID. We were also interested to observe patterns of individual differences in people’s abilities to spot fraudulent (morphed) images. In particular, how does the ability to spot a morph relate to people’s matching performance on unaltered images? It is well-established that there are large individual differences in people’s unfamiliar face matching performance [17], and this is exploited through recruitment of ‘super-recognisers’ by professional groups including London’s Metropolitan Police [19]. However, it is not clear whether individuals who are good (or poor) at face matching would be correspondingly good (or poor) at detecting graphically-manipulated face images. For this reason, we examined the relationship between these two abilities in this experiment.

### Method

#### Ethics statement

This study was approved by the Ethics Committee of the Department of Psychology, University of York. All participants gave written informed consent.

#### Participants

Forty two participants (34 female) with a mean age of 21 years (SD = 3, Range = 18–31) were recruited from the University of York Department of Psychology. All participants were naïve to the purpose of the study and received a course credit or monetary payment for their participation.

#### Procedure

The experimental stimuli, apparatus and procedure used in the present experiment were identical to those described in Experiment 1, with the exception that an extra response option was made available on each trial. Prior to the experiment, participants were given instructions about the morphing technique, and were shown an example sequence of images morphing between faces. They were also instructed that they could spot morphs from image irregularities such as ‘the ghost outline of another face’. They were instructed to examine image pairs in each trial carefully, and to decide whether the person shown in the passport image was the same person as the target (match), a different person (mismatch) or a morphed image.

### Results and discussion

#### Performance levels

**Fig 4:**
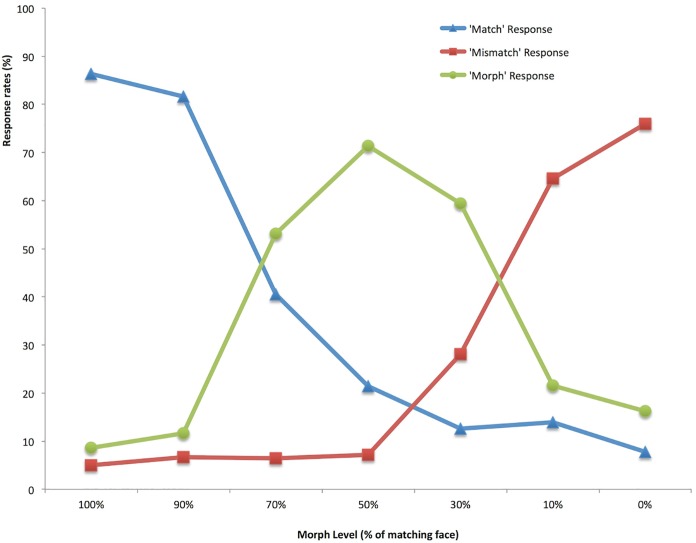
Mean response rates across morph levels for Experiment 2.

Fig 4 shows the mean responses (match, mismatch, morph) given to images containing different levels of the target identity. Error rates for endpoints (100% match and 100% mismatch) are low, and consistent with average population performance on the GFMT (Long version). Two factor ANOVA (Response Type x Morph Level) confirms the interaction which is clear in Fig 4 (F(12,492) = 125.2, p < 0.001, *η*^2^ = .75). Tukey HSD tests performed across Simple Main Effects of Morph Level show the following. For Match Responses, there is no significant difference between 100% and 90%, or in steps between 50%, 30%, 10% and 0. However, there are significant drops in Match responses for each of the steps between 90%, 70% and 50% (F = 117.6 and 25.2 respectively). Furthermore, there is a significant difference in performance for pairwise comparison of 50% and 0% levels (F = 13.0). This pattern is echoed for Mismatch responses: There are no significant differences between 100% and 50% morph levels, but there are significant rises in ‘Mismatch’ responses for each of the steps between 50%, 30%, 10% and 0% (F = 30.2, 93.0 and 8.8, respectively). Morph detection is a clear function of morph level, with maximum detection at 50/50 morph (Fig 4). Tukey HSD comparisons show no significant differences between 100% and 90% or between 10% and 0%, but reliable effects at each step in between (steps from 90% to 10%, Fs = 119.5, 23.2, 9.9, 99.9 respectively).

This pattern shows very marked improvements over Experiment 1. When not briefed about the possibility of ‘morph-fraud’, participants in the first experiment accepted 50/50 morphs as genuine ID at a rate of 68%. However, participants told to expect some morph images, are now accepting 50/50 as genuine at only 21%. This is a very large reduction—though note that it is still far from perfect performance, and remains significantly higher than baseline—the level at which viewers accept a mismatched photo (8% here). So, it appears that prior instructions can greatly improve errors of false acceptance, but not eliminate them entirely.

#### Individual differences

**Fig 5:**
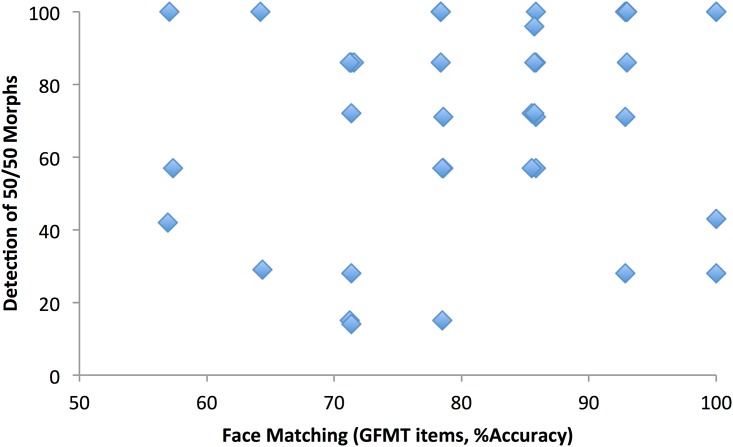
Face matching plotted against proportion of 50/50 morphs detected (some points over-lay).

For each participant, we first calculated an accuracy score based only on the non-morphed images (i.e. simple match or non-match identities). This represents a subset of the items from the GFMT (14 items, 7 match/7 mismatch), and we expect to observe some individual differences in these scores, based on previous research. These scores are plotted in Fig 5, against the participants’ ability to detect a 50/50 morph—i.e. the proportion of times they responded ‘morph’ to these images, across the experiment. It is clear from Fig 5 that there are large individual differences in both these dimensions. In particular, note that there was a huge range in the rate at which individual participants spotted morphs—ranging from 14% to 100%, across the experiment. Pearson correlation shows no significant association between face matching ability and morph-detection (r = .28, N = 42, p = .073).

We next examined associations between morph detection and face matching separately for match and mismatch GFMT items only. Previous work [20] has shown a dissociation between performance on matching and mismatching items, and this was replicated in the current data (*r* = 0.13, N = 42, *p* = 0.38)–though note that this is based on only a small number of items (7 match and 7 mismatch) per participant. The detailed analysis revealed an interesting pattern. There was no association between morph detection and accuracy for matching items (*r* = 0.006, N = 42, *p* = 0.97). However, there was a significant association between morph detection and accuracy for mismatching items (*r* = 0.36, N = 42, *p* = 0.019). In general, people who were good at spotting morphs were also good at spotting that two unmanipulated pictures showed different faces.

This is an interesting result, because it uncovers the component of face matching ability that is associated with fraudulent morph detection. This is relevant for professional bodies recruiting specialist facial analysts (or ‘super-recognisers’), as it demonstrates that the different components involved in checking validity of photo-ID can manifest in different ways. Note that all the participants in this study had received some training, in that they were alerted to graphical ‘fraud’ at the start of the experiment, and had been shown examples. Nevertheless, as can be seen in Fig 5, some participants were able to perform normal unfamiliar face matching at outstanding levels of performance (100% in some cases) while being very poor at detecting these fraudulent images. The 50/50 morph tested here is the most useful for potential fraudsters, as it gives the highest likelihood of being accepted as photo-ID for two different people (Experiment 1). However, it is also the image with the largest amount of graphical change and is generally the easiest to spot (Fig 4). It is therefore very striking that the overall levels of performance incorporate such a vast set of individual differences—differences which add further, independent, complexity to the problem of identity matching.

## Experiment 3

Experiments 1 and 2 show some of the limits of morph detection by human viewers. In this final experiment, we ask whether an automated face-recognition device is vulnerable to morphed ID. Automated face recognition is becoming very common both in high security settings such as border control, and in more everyday settings such as accessing a mobile phone. In previous work [21] we have shown that a face recognition system used to unlock a smartphone, can benefit greatly from some graphical manipulation. In particular, we showed that storing an ‘average’ of the owner’s face, derived from several different images of the same person, can significantly enhance the operation—forming a representation which recognises the user over a wide range of conditions. In this study, we ask whether a smartphone might be susceptible to accepting a morph between two individuals—hence rendering it unable to distinguish the two. To test this, we asked volunteers to try to unlock the device using its built-in face recognition system. We had previously stored an image of this ‘user’ (which should allow access), an image of another person (which should deny access) or an image containing a morph between the two.

### Method

#### Ethics statement

This study was approved by the Ethics Committee of the Department of Psychology, University of York. All participants gave written informed consent.

#### Participants

Twenty participants (18 female) with a mean age of 23 years (SD = 6, Range = 19–43) were recruited from the University of York Department of Psychology. All participants were naïve to the purpose of the study and received a course credit or monetary payment for their participation.

#### Smartphone specifications

A Samsung Galaxy S3 smartphone with an Android operating system (v.4.4.2) was used in the present experiment. The face authentication security feature is part of the standard software package installed on the device. Throughout the experiment, the phone was placed in ‘flight mode’, a feature which disables all wireless functions while retaining full use of the camera. To capture the live face of the user, the recognition algorithm relies on a front-facing two megapixel camera/video recorder (30 frames/s).

#### Stimuli

Photos of each participant were taken with an iPhone-6, 8-mega-pixel camera, in good indoor lighting. These were cropped to 270 x 348 pixels for subsequent use in morphing software. For each participant, we chose a face from the GFMT (i.e. those images used in Experiments 1 and 2), which gave rise to the same general description. This face acted as the ‘mismatching’ person in the tests. (NB—for three participants, it was not possible to choose a corresponding mismatch pair from the GFMT faces, because the database is entirely Caucasian and the volunteer participants were not. For these three faces, we chose a foil by internet search instead. All subsequent analysis was conducted with and without these participants, in case this difference introduced a systematic effect. However, there are no qualitative differences between analyses including or excluding these participants, and so we report the inclusive analysis.)

**Fig 6:**
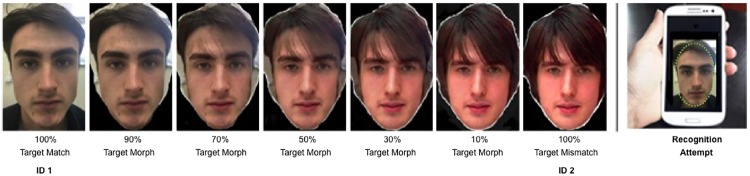
Morph levels between a participant and a foil face from the GFMT. The individuals shown in Fig 6 have given written informed consent (as outlined in PLOS consent form) to publish these images.

Following the same method described for Experiments 1 and 2, Psychomorph was used to create the morphed images for each identity in a pair, resulting in a matching face, a mismatching face, and 5 morphed intermediates containing between 90% and 10% of the target. Fig 6 shows an example.

#### Procedure

Participants were requested to activate the phone’s face recognition software by pressing the ‘power’ button and presenting their face to the inbuilt camera. The smartphone would then either allow or deny the user access. Prior to this, the experimenter had loaded the smartphone with one of the images relevant to that participant, i.e. their own face, the mismatch face chosen for them, or one of the morphs between these two. Participants were always unaware of which photo was stored on the phone for any trial, and the order of images tested was randomised. Testing took place in good lighting, in an office with artificial light and a large window. For each stored image, participants made 10 attempts to open the phone. They were asked to rotate their swivel chairs round the whole 360 degrees across the 10 attempts to open the camera, in order to incorporate naturalistic and unsystematic changes in lighting conditions.

### Results and discussion

**Fig 7:**
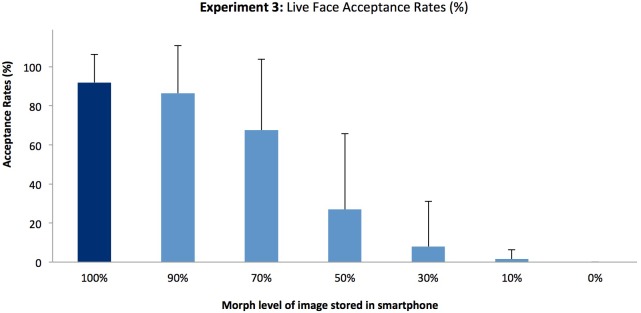
Mean acceptance rates across morph levels for Experiment 3. (Error bars denote standard deviation).

Fig 7 shows acceptance rates for faces at different morph levels. Error rates at endpoints show that the phones are not perfect in giving access to genuine users (91.8% acceptance for the matching image), but never give access to the foil. Single-factor ANOVA across conditions shows a strong influence of morph level (F(6,114) = 78.4, p < .001, *η*^2^ = .80). Tukey HSD comparisons between morph levels show no significant difference in acceptance rates between 100% and 90%, but significant drops between steps 90%, 70% and 50% (F = 14.16 and 39.0 respectively), and between 50% and 0% (F = 17.34).

These results indicate that the automatic face recognition system tested here is relatively safe from morphed identity attacks. The system does accept 50% morphs at a rate of 27%—and while this is perhaps not ideal, the acceptance rate falls well below a level at which two faces would be indistinguishable. In general, the decision ‘strategy’ of the device is conservative—making only denial of entry errors, and never allowing an ‘imposter’ access to the system. This conservative strategy also protects against a potential vulnerability identified in Experiment 1 for novice human viewers.

## General discussion

We have presented three experiments examining the potential of facial morphs for use in fraudulent documents. In each of these we have tested human (Experiments 1 and 2) or smartphone (Experiment 3) recognition across a series of images gradually morphing one face into another. However, the most interesting case across experiments is the 50/50 morph, which contains equal contribution from two faces. This provides a potential route to fraud, as it could be seen as sufficiently similar to each contributing person to serve as photo-ID for both. So, for example, in the case of passports, one person might apply for the document, and another person use it to cross borders.

Our results are generally encouraging—suggesting that this form of attack is unlikely to prove very efficient. Although viewers are willing to accept 50/50 morphs as true ID at worryingly high rates (68%, Experiment 1) it is relatively easy to reduce this error rate significantly (to 21% in Experiment 2) by some simple instructions. Furthermore, for the automated technology tested (a currently popular smartphone) acceptance rates of 50/50 morphs are comparable to trained human perceivers (27% in Experiment 3). While these rates are quite low, they are far from perfect—and always significantly above acceptance of a fraudulent photo of another person. This suggests there may, on some occasions, be benefit to fraudsters in using this approach. However, it is far from being a reliable strategy.

How likely are these results to scale to real world problems? Of course, participants in psychology experiments are certainly not motivated to the level of professionals checking ID in security-critical settings. Furthermore, the minimal training given in Experiment 2 is likely to be very scant by comparison to real settings. However, ID checks are not limited to these critical arenas, and photo matching is a routine part of daily life, for example in retail outlets and bars, where staff sell age-restricted goods. In these settings, it is easily possible that false acceptance rates at the levels described here could be replicated—as they have been in previous studies comparing photo-ID to live targets [5–7].

Experiment 2 also revealed large individual differences in participants’ ability to detect morphs. However, further analysis revealed that this related only to one component of face matching—i.e. a facility to detect mismatching face photos. It has been known for some time that certain aspects of face processing are dissociable; people good at one face task are not necessarily good at another. The dissociation reported here is a further reminder that not all tasks with faces are the same—efficient performance in any applied setting is likely to require a proper task analysis, with personnel assigned optimally to different subcomponents of the job.

Finally, the morph technique used here was relatively unsophisticated. We used a readily available morphing program, with no further ‘touching-up’ of the images (see Figs 2 and 6). It seems entirely reasonable to assume that any serious attempt to produce fraudulent morph images would eliminate some of the image artefacts introduced by this technique—such as the shadowing which occurs towards the middle of the morph sequence. It is therefore perhaps surprising that acceptance rates were as high as they were—and it is possible that these results underestimate the levels of acceptance for realistic attempts at graphical fraud. Policing of ID from face photographs remains a complex process, and each new opportunity for fraud requires psychological, as well as technical, investigation.

## Supporting information

S1 Table Individual subject scores for each of the three experiments. (XLSX)

S1 Text Figure permissions. (DOCX)

## References

[1] UK HM Passport Office Report: Basic Passport Check. 2015. [Cited 2016 Nov 21]. https://www.gov.uk/government/publications/basic-passport-checks.

[2] UK National Fraud Authority Report: Fighting Fraud Together. 2011. [Cited 2016 Nov 21]. https://www.gov.uk/government/uploads/system/uploads/attachment_data/file/118501/fighting-fraud-together.pdf

[3] Bruce V, Henderson Z, Greenwood K, Hancock PJB, Burton AM, Miller P. Verification of face identities from images captured on video. J Exp Psychol Appl. 1999;5(4):339–60.

[4] Megreya AM, Burton AM. Unfamiliar faces are not faces: Evidence from a matching task. Mem Cognit. 2006;34(4):865–76. 17063917 10.3758/bf03193433

[5] Kemp RI, Towell N, Pike G. When seeing should not be believing: Photographs, credit cards and fraud. Appl Cogn Psychol. 1997;11(3):211–22.

[6] Megreya AM, Burton AM. Matching faces to photographs: Poor performance in eyewitness memory (without the memory). J Exp Psychol Appl. 2008;14(4):364–72. 10.1037/a0013464 19102619

[7] Davis JP, Valentine T. CCTV on trial: Matching video images with the defendant in the dock. Appl Cogn Psychol. 2009;23(4):482–505.

[8] Megreya AM, Sandford A, Burton AM. Matching Face Images Taken on the Same Day or Months Apart: the Limitations of Photo ID. Appl Cogn Psychol. 2013;27(6):700–6.

[9] White D, Kemp RI, Jenkins R, Matheson M, Burton AM. Passport Officers’ Errors in Face Matching. PLoS One. 2014;9(8):e103510 10.1371/journal.pone.0103510 25133682 PMC4136722

[10] Ghatol NP, Paigude R, Shirke A. Image Morphing Detection by Locating Tampered Pixels with Demosaicing Algorithms. Int J Comput Appl. 2013;66(8):23–6.

[11] Ferrara M., Franco A and Maltoni D. On the Effects of Image Alterations on Face Recognition Accuracy In: Bourlani T, editor. Face Recognition across the Imaging Spectrum. Switzerland: Springer; 2016 p. 195–222.

[12] Beale JM, Keil FC. Categorical effects in the perception of faces. Cognition. 1995;57(3):217–39. 8556842 10.1016/0010-0277(95)00669-x

[13] Campanella S, Chrysochoos a., Bruyer R. Categorical perception of facial gender information: Behavioural evidence and the face-space metaphor. Vis cogn. 2001;8(2):237–62.

[14] Kikutani M, Roberson D, Hanley JR. What’s in the name? Categorical perception for unfamiliar faces can occur through labeling. Psychon Bull Rev. 2008;15(4):787–94. 18792505 10.3758/pbr.15.4.787

[15] McKone E, Martini P, Nakayama K. Categorical perception of face identity in noise isolates configural processing. J Exp Psychol Percept Perform. 2001;27(3):573. 10.1037//0096-1523.27.3.573 11424647

[16] Kikutani M, Roberson D, Hanley JR. Categorical perception for unfamiliar faces. The effect of covert and overt face learning. Psychol Sci. 2010;21(6):865–72. 10.1177/0956797610371964 20483817

[17] Burton AM, White D, McNeill A. The Glasgow Face Matching Test. Behav Res Methods. 2010;42(1):286–91. 10.3758/BRM.42.1.286 20160307

[18] Tiddeman B, Burt M, Perrett D. Prototyping and transforming facial textures for perception research. IEEE Comput Graph Appl. 2001;21(5):42–50.

[19] Robertson DJ, Noyes E, Dowsett A, Jenkins R, Burton AM. Face recognition by Metropolitan Police Super-recognisers. PLoS One. 2016;11(2, e0150036):1–8. 10.1371/journal.pone.0150036 PMC4769018 26918457

[20] Megreya AM, Burton AM. Hits and false positives in face matching: A familiarity-based dissociation. Percept Psychophys. 2007:69(7): 1175–1184. 18038955 10.3758/bf03193954

[21] Robertson DJ, Kramer RSS, Burton AM. Face Averages Enhance User Recognition for Smartphone Security. PLoS One. 2015;10(3): e01:1–11. 10.1371/journal.pone.0119460 PMC4373928 25807251

