# Supplementary material for: Fraudulent ID using face morphs: Experiments on human and automatic recognition
Source: PLoS One. 2017 Mar 22;12(3):e0173319. doi: 10.1371/journal.pone.0173319 (PMC5362102; doi:10.1371/journal.pone.0173319)
Supplement: S1 Text — (DOCX) [file pone.0173319.s002.docx]

Fig 1. Copyright A.M. Burton (corresponding author), free to publish under the Creative Commons Attribution License (CCAL), CC BY 4.0.

Fig 2. Copyright A.M. Burton (corresponding author), free to publish under the Creative Commons Attribution License (CCAL), CC BY 4.0.

Fig 6. Copyright A.M. Burton (corresponding author), free to publish under the Creative Commons Attribution License (CCAL), CC BY 4.0.
